# The Efficacy of Silicone Gel for the Treatment of Hypertrophic Scars and Keloids

**DOI:** 10.4103/0974-2077.58527

**Published:** 2009

**Authors:** Neerja Puri, Ashutosh Talwar

**Affiliations:** *Department of Dermatology, Guru Gobind Singh Medical College, Faridkot, Punjab, India*

**Keywords:** Keloids, scars, silicone

## Abstract

Topical self drying silicone gel is a relatively recent treatment modality promoted as an alternative to topical silicone gel sheeting. Thirty patients with scars of different types including superficial scars, hypertrophic scars, and keloids were treated with silicon gel application. The results of the self-drying silicone gel have been satisfactory.

## INTRODUCTION

Scars vary greatly in quality, depending on individual and racial patient features, the nature of the trauma, and the conditions of wound healing.[1] They frequently determine aesthetic impairment and may be symptomatic, causing itching, tenderness, pain, sleep disturbance, anxiety, depression and disruption of daily activities. Other psychological sequelae include posttraumatic stress reactions, loss of self esteem and stigmatization leading to a diminished quality of life. Scar contractures also can determine disabling physical deformities.[2‐5] All these problems are more troublesome to the individual patient, particularly when the scar cannot be hidden by clothes. This study was undertaken to verify the efficacy of a new topical silicone treatment; a self-drying spreadable gel that needs no means of fixation and cannot be seen because of complete transparency.

Silicone gel contains long chain silicone polymer (polysiloxanes), silicone dioxide and volatile component. Long chain silicone polymers cross link with silicone dioxide. It spreads as an ultra thin sheet and works 24 hours per day.[6,7] It has a self drying technology and itself dries within 4-5 minutes. It has been reported to be effective and produce 86% reduction in texture, 84% in color and 68% in height of scars.[8,9] Silicon gel exerts several actions which may explain this benefit in scars:

- It increases hydration of stratum corneum and thereby facilitates regulation of fibroblast production and reduction in collagen production. It results into softer and flatter scar. It allows skin to "breathe".
- It protects the scarred tissue from bacterial invasion and prevents bacteria-induced excessive collagen production in the scar tissue.
- It modulates the expression of growth factors, fibroblast growth factor β (FGF β) and tumor growth factor β (TGF β). TGF β stimulates fibroblasts to synthesize collagen and fibronectin. FGF β normalizes the collagen synthesis in an abnormal scar and increases the level of collagenases which breaks down the excess collagen. Balance of fibrogenesis and fibrolysis is ultimately restored.
- Silicone gel reduces itching and discomfort associated with scars.

The advantages of silicon gel include easy administration, even for sensitive skin and in children. It can be applied for any irregular skin or scar surfaces, the face, moving parts (joints and flexures) and any size of scars. A tube of 15 gram contains enough silicone gel to treat 3-4 inches (7.5-10 cm) scar twice a day for over 90 days.

## MATERIALS AND METHODS

The study enrolled 30 patients having scars. Written informed consent was taken from all the patients before the study. Also, prior approval of hospital ethical committee was taken before the study. The silicone gel was applied as a thin film twice a day. It was rubbed with fingertips for 2-3 minutes. For fresh scars, treatment was started just days after wound closure or after 5-10 days. The scars were evaluated at monthly intervals. The appearance of scar, including scar type, scar size and scar color was assessed by the physician. We classified hypertrophic scar as a red or dark pink, raised (elevated) sometimes itchy scar confined within the border of the original surgical incision, with spontaneous regression after several months and a generally poor final appearance. A keloid is instead classified as a scar red to brown in colour, very elevated, larger than the wound margins very hard and sometimes painful or pruritic with no spontaneous regression.

Patients were observed and the results were compared at monthly follow up examinations. Follow up was done for 6 months. All scars were measured and photographed before treatment onset. Scars were graded 1 to 4 on the basis of criteria in Table 1. Final photographs were taken at this time.

**Table 1:** Classification of scars according to morphologic features

Grade	
I (Normal)	Flat, soft, normal color
II (Mildly hypertrophic)	Slightly elevated, moderately hard, light to dark pink color
III (Hypertrophic)	Elevated (within wound margins) hard, dark pink to dark red color
IV (Keloid)	Very elevated, larger than wound margins, very hard, red to brown color

## RESULTS

**Figure 1:**
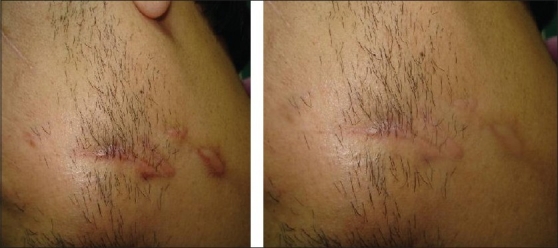
A patient with hypertrophic scar before and after treatment

**Figure 2:**
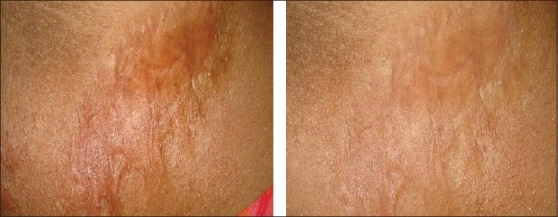
A patient with burn scar before and after treatment

**Figure 3:**
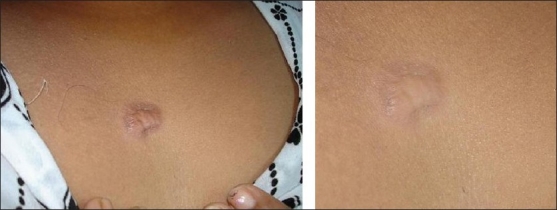
A patient with minor keloid before and after treatment

Eleven cases (36.66%) were in the age groups of 30-40 years, 8 (26.66%) cases between 20-30 years, 5 (16.66%) cases between 40-50 years, 2 (6.610%) cases between 10-20 years and 50-60 years of age each and 1 (3.33%) case of > 60 years of age and between 5 and 10 years of age each. Male:Female ratio was 2:1. It was also seen that most (40%) of the scars were between 1 and 3 months duration, 26.6% of scars were of less than 1 month duration, 20% of scars were between 3 and 6 months and 13.33% scars were of more than 6 months duration. The commonest type of scars were hypertrophic scars (Grade III, 50%) followed by mildly hypertrophic scars (Grade II, 26.6%) and keloids (Grade IV, 23.33%). Most of the scars were between 1 and 3 months duration (40%), 26.6% of scars were of less than 1 month duration, 20% of scars were between 3 and 6 months and 13.33% scars were of more than 6 months duration,

After treatment, improvement was noted in the scars [Figures 1‐3]. Sixty percent scars were graded as normal (Grade I), while 20% were graded as mildly hypertrophic (Grade II). Twenty percent of scars were of Grade III and IV at the end of study;10% in each grade [Table 2]. Side effects were few. Allergic reaction to silicone gel was seen in one case and mild desquamation was seen in 2 cases.

**Table 2:** Grading of scars after treatment

Grading	Number of cases	Percentage
I (Normal)	18	60
II (Mildly hypertrophic)	6	20
III (Hypertrophic)	3	10
IV (Keloid)	3	10

## DISCUSSION

Since the early 1980s, silicone gel sheeting has been widely used in the treatment of hypertrophic scars and keloids. Several clinical studies and reviews have confirmed its efficacy.[10,11]

While many treatments have been suggested in the past for scars, only a few of them have been supported by prospective studies with adequate control group. Only two treatments can be said to have sufficient evidence for scar management; topical application of silicone gel sheeting and the intralesional injection of corticosteroids.[12] The former generally is indicated as both a preventive and the therapeutic device, the latter as a therapeutic agent only.[13] Topical silicone gel sheeting is cumbersome to keep on the scar, and the patient compliance often is low for lesions in visible areas.[14‐16] Tapes or bandaging frequently is not accepted. It may also lead to skin irritation, which can require discontinuation of treatment, especially in hot climates. Gel sheeting is effective for scar control, but patient compliance with the method is not always satisfactory.[17‐19] Steroid injections are painful and may lead to skin atrophy and dyschromies. They usually are contraindicated for large areas and for children.

Topical silicon gel application can overcome some of these limitations.

Self drying silicone gel is appealing because it is effective, no fixation is required; it is invisible when dry; and sun blocks, makeup or both can be applied in combination.

However, on areas of the body covered by clothes, it must be perfectly dry before the patient dresses, and this may not be always practical. All the patients felt the gel was easy to apply, but some complained of prolonged drying time. The use of a hair dryer was recommended to overcome this problem. When the scars are located in visible areas, especially on the face, patients can experience psychological discomfort with the visibility of the treatment. In warm climates, skin reactions are relatively common, often leading to treatment interruption.[20,21]

## CONCLUSIONS

Topical silicon gel is safe and effective treatment for hypertrophic and keloidal scars. It is easy to apply and cosmetically acceptable.

## References

[1] Tuan TL, Nichter LS (1998). The molecular basis of keloid and hypertrophic scar formation. Mol Med Today.

[2] Dyakov R, Hadjiiski O (2000). Complex treatment and prophylaxis of post-burn cicatrization in childhood. Ann Burns Fire Disasters.

[3] Ahn ST (1989). Topical silicone gel: A new treatment for hypertrophic scars. Surgery.

[4] Ahn ST (1991). Topical silicone gel for the prevention and treatment of hypertrophic scar. Arch Surg.

[5] Beranek JT (1990). Why does topical silicone gel improve hypertrophic scars? A hypothesis. Surgery.

[6] Quinn KJ (1987). Silicone gel in the scar treatment. Burns.

[7] Sawada Y, Sone K (1990). Treatment of scars and keloids with a cream containing silicone oil. Br J Plast Surg.

[8] Poston J (2000). The use of the silicone gel sheeting in the management of hypertrophic and keloids scars. J Wound Care.

[9] Suetake T, Sasai S, Zhen YX, Tagami H (2000). Effects of silicone gel sheet on the stratum corneum hydration. Br J Plast Surg.

[10] Baum TM, Busuito MJ (1998). Use of glycerine-based gel sheeting in scar management. Adv Wound Care.

[11] Niessen FB (1998). The use of silicone occlusive sheeting (Sil-K) and silicone occlusive gel (Epiderm) in the prevention of hypertrophic scar formation. Plast Reconstr Surg.

[12] Mustoe TA, Cooter RD, Gold MH, Hobbs FD, Ramelet AA, Shakespeare PG (2002). International clinical recommendations on scar management. Plast Reconstruc Surg.

[13] Berman B, Flores F (1999). Comparison of a silicone gel filled cushion and silicone gel sheeting for the treatment of hypertrophic or keloid scars. Dermatol Surg.

[14] Carney SA, Cason CG, Gowar JP (1994). Cica care gel sheeting in the management of hypertrophic scarring. Burns.

[15] Perkins K, Davey RB, Wallis KS (1983). Silicone gel: A new treatment for burn scars contractures. Burns Incl Therm Inj.

[16] Mercer NS (1989). Silicone gel in the treatment of keloid scars. Br J Plast Surg.

[17] Dockery GL (1994). Treatment of hypertrophic and keloid scars with silastic gel sheeting. J Foot Ankle Surg.

[18] Cruz-Korchin NI (1996). Effectiveness of silicone sheets in the prevention of hypertrophic breast scars. Ann Plast Surg.

[19] Gibbons M, Zuker R, Brown M, Candlish S, Snider L, Zimmer P (1994). Experience with silastic gel sheeting in pediatric scarring. J Burn Care Rehabil.

[20] Borgognoni L, Martini L, Chiarugi C, Gelli R, Reali UM (2000). Hypertrophic scars and keloids: Immunophenotypic features and silicone sheets to prevent recurrences. Ann Burns Fire Disasters.

[21] Nikkonen MM, Pitkanen JM, Al Qattan MM (2001). Problems associated with the use of silicone gel sheeting for hypertrophic scars in the hot climate of Saudi Arabia. Burns.

